# Brain Metastasis in Bone and Soft Tissue Cancers: A Review of Incidence, Interventions, and Outcomes

**DOI:** 10.1155/2014/475175

**Published:** 2014-03-16

**Authors:** Faris Shweikeh, Laura Bukavina, Kashif Saeed, Reem Sarkis, Aarushi Suneja, Fadi Sweiss, Doniel Drazin

**Affiliations:** ^1^College of Medicine, Northeast Ohio Medical University, 4209 State Route 44, Rootstown, OH 44272, USA; ^2^Department of Medicine, Summa Health System, Akron, OH 44303, USA; ^3^Johns Hopkins School of Public Health, Baltimore, MD 21205, USA; ^4^Department of Physical Medicine and Rehabilitation, Rush University Medical Center, Chicago, IL 60612, USA; ^5^Department of Neurosurgery, George Washington University, Washington, DC 20037, USA; ^6^Department of Neurosurgery, Cedars-Sinai Medical Center, Los Angeles, CA 90048, USA

## Abstract

Bone and soft tissue malignancies account for a small portion of brain metastases. In this review, we characterize their incidence, treatments, and prognosis. Most of the data in the literature is based on case reports and small case series. Less than 5% of brain metastases are from bone and soft tissue sarcomas, occurring most commonly in Ewing's sarcoma, malignant fibrous tumors, and osteosarcoma. Mean interval from initial cancer diagnosis to brain metastasis is in the range of 20–30 months, with most being detected before 24 months (osteosarcoma, Ewing sarcoma, chordoma, angiosarcoma, and rhabdomyosarcoma), some at 24–36 months (malignant fibrous tumors, malignant peripheral nerve sheath tumors, and alveolar soft part sarcoma), and a few after 36 months (chondrosarcoma and liposarcoma). Overall mean survival ranges between 7 and 16 months, with the majority surviving < 12 months (Ewing's sarcoma, liposarcoma, malignant fibrous tumors, malignant peripheral nerve sheath tumors, angiosarcoma and chordomas). Management is heterogeneous involving surgery, radiosurgery, radiotherapy, and chemotherapy. While a survival advantage may exist for those given aggressive treatment involving surgical resection, such patients tended to have a favorable preoperative performance status and minimal systemic disease.

## 1. Introduction

It is estimated that up to 30% of patients with cancer will develop brain involvement [1, 2]. Breast cancer, nonsmall cell lung cancer, and melanoma have shown a predilection for brain metastasis [3, 4]. Brain metastases are 10 times more common than primary brain tumors, resulting mostly from carcinomas [1–4]. The incidence is much lower in the pediatric population with estimates of 1.5–2.5% [3].

Accounting for 0.8% of all cancers, musculoskeletal bone and soft tissue sarcomas make up a small portion of patients with brain metastases [4]. It is estimated that 3% of all brain metastases are sarcomas and 1–8% of all sarcoma patients may develop brain involvement [1, 2]. However, there is mounting evidence that the incidence is increasing due to new chemo- and radiotherapeutic treatments that prolong survival through systemic disease control but without effective intracranial control [2, 5]. Unlike many other brain metastases, sarcomas tend to be highly radio- and chemoresistant with surgical resection as the basis for management [1]. Mechanisms of sarcoma spread to the brain are twofold: hematogenous dissemination into brain parenchyma and contiguous extension of metastases in bones of the skull into intracranial structures [6].

The purpose of this review is to present the current literature on brain metastasis (BM) from bone and soft tissue cancers, with an emphasis on musculoskeletal sarcomas and those most commonly metastasizing to the brain. We describe the incidence, diagnostic strategies, treatment paradigms, and prognostic outcomes as well as relevant background information.

## 2. Methods

A search of the published literature was conducted for patients with brain metastasis from musculoskeletal sarcomas. The national library of medicine search engine, PubMed, was utilized for the literature search. For each of the sarcomas, the search terms “brain” and “intracranial” were combined with the tumor's name: “osteosarcoma,” “Ewing's sarcoma,” “chondrosarcoma,” “chordoma,” “malignant fibrous tumor,” “malignant fibrous histiocytoma,” “fibrosarcoma,” “liposarcoma,” “alveolar soft part sarcoma,” “rhabdomyosarcoma,” “malignant peripheral nerve sheath tumor,” “MPNST,” or “angiosarcoma.” Relevant articles describing case reports or clinical studies were selected, and the reference lists from these articles were also inspected for other relevant articles. Each of the resultant articles were examined closely and reported in this review. Cases in which there was contiguous extension of a primary tumor into intracranial structures (i.e., a skull tumor) as opposed to frank metastasis from a distant site were excluded. Only publications in English, peer-reviewed journals were included.

## 3. Results

### 3.1. Malignant Bone Tumors

The primary malignant bone tumors discussed are the most commonly reported to metastasize to the brain: osteosarcoma (osteogenic sarcoma), Ewing's sarcoma, chondrosarcoma, chordoma, and fibroblastic/fibrohistiocytic tumors.

#### 3.1.1. Osteosarcoma (Osteogenic Sarcoma)

After plasmacytoma (33%), osteosarcoma (20%) is the most frequently occurring primary malignant bone tumor [7], characterized by osteoid producing atypical cells [8]. In children, osteosarcoma is the most common bone cancer [9]. Dissemination is typically via the bloodstream, primarily targeting lungs and other bones [10]. BM is rare, with a reported incidence of 1.8–5.6% [3], and associated with prior pulmonary metastasis [11], with the hypothesis of lung tumor emboli invading the brain. Nonetheless, there are several reports of BM without active lung involvement. An increased risk of BM with metastatic disease at presentation or with recurrence at 1 year has been reported [3]. As in other BM, those from osteosarcoma typically locate through the anterior circulation to the gray-white matter junction [12]. Multimodality treatment is often involved, though no consensus on treatment exists.

Table 1 summarizes published cases of osteosarcoma patients with BM [2–5, 9, 13–28]. A total of 55 patients are presented, with an average age of 18 years and a male: female ratio of 3 : 2. Location of the primary was variable, with majority localized to the femur. BM management almost always involved surgical resection (SR). This was followed by whole brain radiation therapy (WBRT) and/or chemotherapy in select cases. Mean interval to BM from initial diagnosis (IB) was approximately 18.9 ± 21.1 months (range 0–110) from diagnosis of the primary, and overall mean survival (OS) was approximately 18.4 ± 30.4 months following its detection. A possible predilection for synchronous metastasis has also been highlighted [13, 14]. Location of metastatic lesions varied throughout the cerebrum with the frontal lobe being the most common single lesion.

Yonemoto et al. [18] recommended performing brain imaging periodically in patients with known active pulmonary metastasis. This was echoed by Marina et al. [19] for those with metastatic disease at diagnosis or in whom recurrence develops within 12 months, though whether routine imaging will improve outcomes is debatable [3]. Though surgery was previously advocated only in patients with solitary BM without systemic disease, more recently concurrent systemic disease has been suggested not to be a contraindication for SR [15, 16]. Paulino et al. [17] reported RT to slow neurological deterioration in their cohort of pediatric patients. Unlike the majority of the authors, Flannery and colleagues [15] utilized Gamma Knife Stereotactic Radiosurgery (GKSRS) in their management and noted the modality to be a viable alternative to surgery in select cases.

As a commonly diagnosed musculoskeletal cancer in children and young adults, osteosarcoma, when metastatic, mainly spreads to the lungs and other bones and rarely to the brain. As such, it is difficult to form consensus guidelines on treatment once BM occurs. As many of these lesions are solitary, surgical excision has been the standard of care, with chemotherapy and RT for palliative measures.

#### 3.1.2. Ewing's Sarcoma

Ewing's sarcoma is commonly seen in young adults and has a slight male predominance [29–31]. Up to 80% of patients have subclinical metastases at time of presentation [29]. It comprises about 10% of primary malignant bone tumors and, like osteosarcoma, the lungs and distant skeletal tissues are common metastatic sites [31]. CNS metastases have been reported in 32–56% of cases, frequently a result of direct extension of bony metastases in close proximity, with BM making up <1.8% of cases [32]. Extremity tumors generally have better prognosis than axial skeleton primaries, which are arduous to completely excise [31–33]. BM usually appears as part of systemic disease, with more than 85% having lung involvement [33].

Approximately 40 cases of ESBM have been described in the literature (Table 2) [2, 4, 9, 15–17, 22, 26, 30, 32, 34–38]. Average age of patients is approximately 20.9 years with >65% males. The majority of BM is localized to the parietal lobe (16) 41%, 12 (31%) to frontal lobe, and 5 (13%) to temporoccipital lobes. IB was 23.2 ± 27.1 months (range: 0–115), with OS of 7.1 ± 7.7 months (range: 0–24) following detection. A combination of treatments was utilized in 17 patients (44%), with 22 given chemotherapy (56%). Reportedly, concomitant use of chemotherapy has increased long-term survival rates to 50–70% [29]. Specific treatments for BM included SR (25%), WBRT (70%), GKSRS (17.5%), and conservative management (17%). Primaries originated most commonly from lower extremities and were generally treated with RT and SR, with SR mostly utilized for axial skeleton tumors.

Overall, the appearance of BM signifies a poor prognostic outcome in patients with ES. The current increased frequency of CNS metastases has been linked to prolonged survival and alteration of host response following chemotherapy [26]. Moreover, recent therapies for metastatic ES have not substantially improved outcomes since the initiation of multidrug chemotherapy [29, 34]. As one of the more common sarcomas metastasizing to the brain, it is hoped that future research may herald improved treatments.

#### 3.1.3. Chondrosarcoma

Chondrosarcoma is a malignancy of mesenchyme that is a common primary bone tumor subsequent to osteosarcoma in frequency [39]. It has been classified based on histological appearance into slow growing, benign grade I to malignant grade III [39, 40]. Distant metastases account for 10% of grade II and 71% of grade III [40], commonly occurring to lungs, other bones, and liver, resulting in a mere 5-year survival of 18% [39]. Primary intracranial chondrosarcoma constitutes only 0.16% of all intracranial tumors. BM is exceedingly rare with only few documented cases [41]. Due to their sporadic nature, treatment options are extrapolated to other sarcomatous metastases.

Table 3 summarizes 12 previous chondrosarcoma patients with BM and shows an average age of 43.4 years, with 6 males and 6 females (50%) [15, 39–48]. There were 2 children (age 18 and younger). Locations of the primary tumor were variable with 6 cases in the extremities (50%) and most often treated with SR or amputation. BM was variable in location and occurred with an IB of 7.9 ± 10.6 years (range: 0.5–34). OS was 16.9 ± 16.7 months (range: 2–44) after BM. Most common treatments were SR in 4 cases (33.3%) and GKSRS in 3 cases (25%). Out of 6 cases in which cause of death was specified, 3 expired due to progressive neurologic disease and 3 expired from systemic disease.

Treatment with chemo- and radiotherapy has resulted in poor results, postulated due to the resistant extracellular matrix, low mitotic index, and sparse vascularization of cells [39, 42]. As such, SR was utilized in most of the documented cases, though outcomes were dismal (Table 3). Flannery et al. [15] reported their experience with GKSRS in 21 patients with 60 sarcomatous BM, 2 from chondrosarcoma. Overall, the treatment resulted in a local tumor control rate of 88% with a median survival of 16 months following BM diagnosis. Tsutsumi et al. [40] also reported their experience with GKSRS in a 60-year-old male with chondrosarcoma BM that resulted in adequate control for over 10 months. Thus, GKSRS can be an effective option for these BM, especially small lesions.

Though chondrosarcoma accounts for 40% of primary bone cancers in adults [42], it rarely metastasizes to the brain but it can occur many years down the line. Once BM is diagnosed, it is usually treated with excisional surgery because of resistance to chemotherapy and radiotherapy [40]. More recently, GKSRS has shown promise to be an effective modality with long-term survival and improvement in quality of life.

#### 3.1.4. Chordoma

Chordomas are rare, malignant tumors, which arise from the embryonic notochord [49–51]. Their indolent and progressive course often correlates with a poor prognosis in which they silently expand for years without any clinical symptoms [49, 50]. They are quite uncommon, representing 4% of all malignant tumors of bone [51] and less than 1% of spine tumors [52]. Only about 70 metastasizing cases have been reported, characterizing chordomas as more locally invasive and less susceptible to distant metastasis [51]. There is a male predominance overall, though sacrococcygeal chordomas are more frequent in females [53]. No age preference is apparent, with children as young as 2 and adults in their 70 s afflicted [50]. The pediatric population, however, has been reported to harbor an aggressive form [52]. While the most common site of presentation is the caudal and cranial poles of the spine [54], chordomas are generally characterized into one of three principal clinicoanatomical categories: cranial, vertebral, and sacrococcygeal [49]. According to Hall and Clark, 50% of patients have sacrum or coccyx involvement followed by 33% with clival involvement [51].

Inherently, chordomas destroy and replace bone in which they develop [55]. Until recently, it was thought that it spreads via direct extension, suggesting frank metastasis seldom occurs [54]. However, latest literature explores many cases of metastasis—especially in those presenting at sacrococcygeal sites. It is estimated 25–43% of sacrococcygeal chordomas will present with subsequent metastasis [51], most often to lymph nodes, liver, and lungs [54]. Metastasis presenting in the brain remains rare [51], and complete resection of the primary is vital for prevention and overall patient survival [53]. Table 3 outlines characteristics from published case reports on BM from chordoma [49, 51–56]. Though SR and RT were initiated, most patients died shortly after brain involvement.

Metastasis from chordomas depends on many factors such as histology, mitotic activity, and treatment regimens [53]. Due to their rarity, optimal treatment remains with disappointing results. Surgical excision, while still the recommended treatment course for cure, is frequently not possible [55] though it does provide a palliative measure to reduce tumor load for subsequent chemotherapy [52]. The challenge continues as chordomas are also relatively radio- and chemoresistant [51, 55]. Further research is vital in order to discover satisfactory alternatives for appropriate therapy.

#### 3.1.5. Fibroblastic and Fibrohistiocytic Tumors

Previously designated under “malignant fibrous tumors,” these are rare malignancies characterized by pleomorphic, high-grade tumor cells with histiocytic, fibroblastic, and myofibroblastic features [57–59]. “Malignant fibrous histiocytoma (MFH)” has been renamed “undifferentiated pleomorphic sarcoma (UPS)” in the most recent WHO Classification of tumors of soft tissues and bone [58]. They rarely occur in children [59] and the predominant affected population is typically adult males [60]. Most common primary tumor sites are the extremities and retroperitoneum [57–60]. There is an exceedingly high reoccurrence rate, with some studies reporting as high as 44% [57]. The prognosis is generally poor with a 42% risk of distant metastasis [57, 61, 62]. Sites of metastasis include lungs, liver, bone, and, very rarely, brain [57, 59, 63, 64].

Although these tumors can originate from either bone or soft tissue, defining the source is difficult [65]. Successful SR of the primary site with clear margins can be difficult; thus, local recurrence and metastasis are frequent. Both soft tissue and bone forms have the propensity for BM as the incidence at autopsy has been reported at 1.5% [64, 66]. Patients experiencing BM remain asymptomatic prior to events such as intracranial hemorrhage (ICH), which has been shown to occur [61, 67, 68]. Prognosis after detection of BM varies significantly and can be affected by the primary site, with soft tissue locations being more susceptible to multiple metastases [69].

As with other sarcomas, pulmonary metastasis is most common, with its detection increasing the risk of future BM [66, 68]. Thus, both the primary site and the site(s) of metastasis are essential in accurate prognosis of patients. As shown in Table 4, the majority were MFH/UPS (17, 54.8%), with the rest being fibrosarcomas (10, 32.3%) and dermatofibrosarcomas (3, 9.7%) [2, 5, 33, 38, 42, 57–73]. Multimodality treatment was utilized in many cases, including SR, chemotherapy, WBRT, and STRS. The IB was 33.2 ± 41.0 months ranging from 0 to 312 months. OS also was variable with a mean of 10.6 ± 17.1 months following BM, and a male predilection was observed (55.6%).

Periodic imaging of the brain, the primary site, and the metastatic sites has been suggested to prolong life expectancy, though it remains controversial [64]. In addition, as these metastases tend to bleed, many patients often become symptomatic only after ICH, further reiterating the significance of routine screening in susceptible patients [61, 64, 67]. Similarly, the therapeutic range of chemotherapeutic agents must be monitored in an organ-specific manner for maximal efficacy [63]. Although cases of BM are uncommon, vigilance is warranted, particularly through frequent neurological examination in all patients and routine imaging in select cases [68].

### 3.2. Malignant Soft Tissue Tumors

The primary malignant soft tissue tumors (STS) discussed are also the most likely to metastasize to the brain: liposarcoma, rhabdomyosarcoma, malignant peripheral nerve sheath tumor, angiosarcoma, and alveolar soft part sarcoma.

#### 3.2.1. Liposarcoma

Liposarcomas comprise about 10–20% of all STS [74–76]. While uncommon in children, liposarcoma is currently the second most frequent soft-tissue malignancy in adults [75]. Middle-aged and older adults are most susceptible [74], presenting most frequently deep within soft tissues of proximal extremities and retroperitoneum as large, bulky masses [74, 76, 77]. The incidence of BM after liposarcoma diagnosis is exceedingly rare and is most often preceded by pulmonary metastasis [75, 77].

Espat et al. [78] analyzed the cohort of 3829 patients from the Memorial Sloan Kettering Cancer Center between 1982 and 1999 who presented with STS. Forty patients (>1%) presented or developed STS BM, 5 (12.5%) of which were liposarcoma patients—the second leading tumor causing STSBM in the cohort. Previous liposarcoma patients with BM are summarized in Table 5 [2, 74–77, 79–82]. Average patient age is 52.3 years. Parenchymal involvement included the temporoparietal region (44%), frontal region (22%), parafalcine (11%), and skull base (11%). The majority originated in the thigh and most patients received SR with or without chemotherapy and/or RT. The same is appreciated for the BM; SR was offered to most, combined with another modality for a few. IB was 127.5 ± 124.0 months and ranged from 0 to 313 months. Survival following BM is unfavorable even after SR, and the use of chemo- and radiotherapy is questionable at best.

As the lifespan of patients diagnosed with liposarcoma extends, the natural history of the disease leads to CNS metastases, which occurs in a large part after 10 years following original diagnosis [2, 76, 79]. Histologically, the degree of differentiation also contributes to the likelihood of metastasis [80]. Kumar and Teasdale [81] noted that the myxoid type liposarcomas have the lowest rates of BM while pleomorphic types have the highest [75, 82]. Additionally, the origin of the liposarcoma dictates the extent of relapse [74, 78]. Regardless of the available treatments, BM from liposarcomas is very unusual and should only be considered after relevant neurological findings [2, 74].

#### 3.2.2. Rhabdomyosarcoma

Rhabdomyosarcoma (RMS) is a malignancy of striated muscle and one of the common STS in those under 20 years [83]. It usually arises from the head and neck, genitourinary system, and the extremities [84]. BM from RMS is exceedingly uncommon and is accompanied with neurologic symptoms in a minority of patients [84, 85]. In his 1988 review, Lewis reported the detection of pulmonary metastases from RMS may indicate an increased risk of future BM [66]. Common metastatic sites are lungs, pleura, pancreas, and bones and occurs via blood or lymph flow [83, 86].

Vezeridis et al. [87] reviewed 242 patients with recurrent STS between 1960 and 1978; 68 (28.1%) were metastatic RMS. In a Japanese study with 480 sarcoma patients [88], 2 of the 13 with metastatic RMS had BM (15.4%). Parasuraman et al. [35] documented patients treated at St. Jude Children's Hospital from 1962 to 1998 with BM from RMS. Out of 419 patients, 10 developed BM (2.4%). Median interval between primary tumor and diagnosis of BM was 1 year. The authors also showed that combined RT and chemotherapy prolong survival and improve prognosis, with an estimated 1-year survival of 30%. Another study reported RT alone was inadequate while intensive chemotherapy and high dose RT to the primary site had a survival of 74% with a local control rate of 89% [89].

Like other sarcomas, multimodality treatment is instigated for those with BM from RMS [83]. Gasparini et al. [86] tracked two series of children with head and neck RMS and intracranial involvement. They found that CNS prophylactic chemotherapy and higher doses of RT resulted in a better ability to achieve persistent local tumor control. As shown in Table 5, previously reported cases of RMS with BM [2, 3, 15, 17, 33, 35, 68, 83–85, 88, 90, 91] have an average age of 12.0 years, with majority being males (21, 56.8%). Most were children (89.2%) including 5 neonates. Thirteen tumors originated from the extremities (35.1%) and treatment of the primary was multimodal. BM were in variable locations including 7 parietal (18.9%), 7 frontal (18.9%), and 5 cerebellar (13.5%). IB is 1.7 ± 2.3 years (range: 0–12.2) and OS was 15.6 ± 16.0 months (range: 0–207). Treatments included WBRT in 20 cases (54.1%), chemotherapy in 18 (48.6%), and SR or GKSRS in 8 (21.6%). Cause of death was neurologic deterioration in 12 (32.4%) and systemic disease in 10 (27.0%), with the rest alive at last followup.

RMS is a common STS affecting young patients, and it has been known to metastasize to different organs and tissues. Patients with a history of metastasis have a significant risk of future BM [3]. Surgical treatment for select patients can result in long-term survival [87, 88], and increasing doses and volume of RT have been shown to prolong survival [86]. Overall, a combined modality approach is advocated.

#### 3.2.3. Malignant Peripheral Nerve Sheath Tumors

A number of entities are synonymous with malignant peripheral nerve sheath tumors (MPNST) including neurofibrosarcoma, neurogenic sarcoma, and malignant schwannoma [92]. MPNST usually arise from nerves of extremities and trunk or from preexisting neurofibromas and account for less than 10% of all STS [93, 94]. Uncommon in the general population with an incidence of 0.001%, it is much more common in those with neurofibromatosis type 1 (NF-1) [93]. Surgery is the primary treatment of MPNST with the aim of radical en bloc SR [93, 95], followed by RT for local control whether clear surgical margins are achieved or not [93, 96, 97]. Due to its infrequency, outcomes are difficult to determine, though reportedly poorer than other sarcomas [92, 98]. The incidence of BM is exceptionally rare with only 21 documented cases (Table 6) [15, 92–94, 99–115].

According to Table 6, MPNST patients with BM have an average age of 36.6 years, with 2/3 males and 4 (19.0%) children. Location of the primary neoplasm varies and its treatment almost always involves surgical resection (SR). The frontal lobe was the most common location for BM (28.6%) and the infratentorial region was involved in 6 cases (28.6%). IB occurred at 32.4 ± 54.5 months (range: 0–180) and OS was 9.9 ± 15.2 months (range: 1–16) following BM. Additionally, at mean follow-up of 42.6 months, 2 patients (10.5%) remained alive. Seven (33.3%) had a history of NF-1. The majority of cases expired due to widely metastatic disease.

Aggressive SR followed by WBRT is the basis of treatment for BM. However, because of the limited pool of patients, there are cases surviving for many months without treatment and others dying over a short period after aggressive intervention [93, 94, 99]. Tilgner et al. [92] reported on a patient with 2 BM treated by WBRT and SR of only one of the lesions. Interestingly, there was local control of both lesions at 14 months, highlighting the importance of WBRT for MPNST BM. The importance of RT should not be underestimated, as one reported case with cerebellar metastasis was treated with en bloc SR without RT only to have local recurrence 4 months later [94]. Another report presented GKRS as a viable option for multiple BM with excellent initial and long-term response [100]. Park et al. [101] noted the tumor's tendency to bleed on presentation and our review builds on this as 5 (23.8%) of all reviewed cases had ICH on presentation (Table 6). Cerebrospinal fluid dissemination is hypothesized as the likely route [102], though hematogenous metastasis, cannot be excluded.

MPNST is a very aggressive cancer associated with high recurrence and metastatic potential. Prognosis is generally poor and worse with metastasis [102, 103, 116]. It does have the potential for BM, though reported mainly in case reports. A greater potential for BM may exist from spinal tumors (Table 6). Treatment should involve aggressive SR combined with RT. Overall, the appearance of BM signifies an impending poor outcome in patients with MPNST. More cases and studies should be reported to help establish a suitable therapeutic approach.

#### 3.2.4. Angiosarcoma

Angiosarcoma is an exceedingly rare malignancy, which arises from endothelial cells of the vasculature. The least common among vascular tumors, angiosarcoma has a prevalence of <1% of all sarcomas [117, 118]. Certain described risk factors include vinyl chloride exposure, chronic lymphedema, and arsenic exposure [119]. They occur mainly in the head, face, liver, skin, and other soft tissues. Most skin and soft tissue angiosarcomas are treated with SR along with adjuvant chemotherapy or RT [118]. BM is unusual, with only a few reported cases (Table 6).

Current understanding of the metastatic behavior of angiosarcomas is limited. Of reported cases with BM, the often involved primary site is the heart [120]. Though, in a retrospective analysis of primary neoplasms of the heart only 2% were angiosarcomas [119]. Review of literature also showed 6 cases of metastatic splenic angiosarcoma, 2 with BM. Table 6 [32, 117–129] shows an average age corresponding to 42.3 years with 70% males. Origin was overrepresented by thoracic or abdominal angiosarcomas (66.6%). Treatment involved SR 93.3% of the time (14/15) and localized RT in only 2/15 cases (13.3%). IB was approximately 16.4 ± 21.7 (range: 0–72) and OS was 4.8 ± 7.3 months, following conservative palliative treatment in 7 (46.6%) and SR in another 7 (46.6%). Metastases were reported mostly in the parietal (30.7%) or frontal lobe (23%). Most expired secondary to systemic complications.

Overall, the prognosis of angiosarcoma is grim. Typical 5-year survival is 12%, with metastases and reoccurrence typically occurring within the first 2 years [118]. Prognosis varies highly with the primary site [122], and surgical resection is often difficult because of hefty tumor size at presentation [119]. Once angiosarcoma has metastasized, especially to the brain, mean survival is decreased to 2–6 months, signifying a bleak outcome.

#### 3.2.5. Alveolar Soft Part Sarcoma

Alveolar soft part sarcomas (ASPS) account for a small percentage of STS at roughly 1% [88, 89]. In contrast to bone-part tumors, ASPS is a soft-part tumor that presents mainly in muscle and deep soft tissue of the thigh or leg [130–133]. A malignancy found in younger patients, those diagnosed are mostly below the age of 40 years [130, 131, 133, 134]. While it metastasizes to lung and bone, unlike other sarcomas, it has a predisposition towards BM with estimates of 15 to 30% in those with stage IV disease [1, 135]. On the other hand, ASPS patients have been reported to have greater oncological control with better relapse-free survival [1]; even though 25% of patients have metastasis at presentation, overall survival is comparatively high at 10–12 years [136, 137].

Portera et al. [134] studied a large cohort of 74 patients with ASPS. The majority (65%) presented with Stage IV disease and BM (19%) was only present in those with other metastases, particularly lung. Eight (88.9%) of the 9 patients with BM developed neurological symptoms, and imaging performed in those without such symptoms did not reveal occult BM. Resistance to conventional chemotherapy was noted and overall survival was 40 months. Daigeler et al. [130] described similar result in their small series and recommended SR followed by RT for both the primary and resectable metastases and found tumor size at presentation to not influence long-term results.

The literature is abundant with reports of BM from ASPS.  Table 7 presents a summary of cases from published series [2, 4, 5, 15, 38, 130, 134, 138, 139]. The IB ranged from 1 to 156 months, roughly averaging 29 months. Patient's age was approximately 30 years, without a remarkable predilection to any one of the sexes. OS ranged significantly (1–36 years), with a mean between 3 and 4 years [130]. Five-year survival reportedly ranges between 59 and 67% [133]. Long-term survivors have been known to exhibit spontaneous regression phenomenon, in which there is partial or complete disappearance of the malignancy in absence of traditional treatment [131].

Neurosurgical resection can be favorable and may increase long-term survival [1, 132, 133, 135]. A surgical advantage may exist with ASPS when compared to other sarcomas. There is little evidence to support routine brain imaging without metastatic disease at other sites. Chemotherapy has not been found to be helpful, as there is frequently lack of response. Even though ASPS has a larger predisposition for BM, patients with ASPS BM generally have a better prognosis than other bone and soft tissue malignancies [1].

#### 3.2.6. Other Soft Tissue Sarcomas

Malignant histiocytomas (undifferentiated pleomorphic sarcomas) and fibrosarcomas originating from the soft tissues are covered under the* fibroblastic and fibrohistiocytic* tumors section. Also, Table 4 includes previous such cases originating from soft tissues with BM. Brain metastases from leiomyosarcomas are frequently reported; however, they rarely originate from musculoskeletal structures. For instance, Salvati et al. [2] have reported 7 cases of brain metastasis from leiomyosarcoma. Most of these cases had their primary site as the uterus, with others reporting ovarian and gastrointestinal origins [3]. Others have reported BM from gastrointestinal origins such as GIST [140].

## 4. Discussion

Previous landmark studies on the treatment of brain metastases have shown efficacy in surgical resection and postoperative WBRT [141, 142], as well as stereotactic radiosurgery [143]. Most such studies have focused on common sources of BM such as lung, breast, and genitourinary cancers. Here, via a thorough literature search, we presented an up-to-date and comprehensive review of the current literature regarding BM from bone and STS. The literature on this topic is limited to case reports and case series. Consequently, variable results are reported with regard to prevalence, management, and outcomes. A few points are noteworthy, however.

Generally, BM from these cancers is very infrequent, most commonly occurring in Ewing's sarcoma, fibroblastic/fibrohistiocytic tumors, and osteosarcoma. Pediatric patients are more likely to have RMS. Though ASPS commonly metastasizes to the brain, it is a much rare cancer and thus accounts for a lesser proportion. Many of the larger studies suggest a slight preponderance in males (52–60%) [2, 5, 9, 38, 144]. It is a disease of younger patients with an average age range between 12 and 52 years and a mean of 32.5. The most important factor in developing BM in many of these cancers is a history of pulmonary metastasis. Most common locations for metastasis follow the anterior circulation, hence, the cerebral hemispheres. Additionally, they tend to be single lesions (>60%), rather than multiple [2]. The point at which BM occurs in these patients is unpredictable, with many found at presentation and others developing many months or even years later. An estimate in the range of 20–30 months is suggested by the literature [2, 5, 9, 38, 144], with most being detected before 24 months (osteosarcoma, Ewing sarcoma, chordoma, angiosarcoma, and rhabdomyosarcoma), some at 24–36 months (fibroblastic/fibrohistiocytic tumors, malignant peripheral nerve sheath tumors, and alveolar soft part sarcoma) and a few after >36 months (chondrosarcoma, liposarcoma). Liposarcoma and chondrosarcoma have repeatedly been reported to develop BM many years following initial diagnosis.

Comprehensive neurological evaluation and imaging should be performed on development of suspicious neurological signs or symptoms. Clinical presentation of BM is characterized by rapid onset of neurological symptoms, such as paresthesias, visual field defects, and headache. Generally, routine neuroimaging studies without these indications are not warranted because of the rarity of BM and because they almost always result in signs and symptoms if they are present though it has been suggested to screen patients with osteosarcoma and MFT with extensive disease, especially if there is a short disease free interval [18, 19, 64].

The extent of systemic disease and Karnofsky Performance Scale (KPS), as well as sarcoma histotype are important factors in determining patient outcomes. The presurgical KPS gives an overall clinical picture, and a score above 60–70 has been cited as a good prognostic indicator associated with higher median survival [2, 5, 9, 38, 144]. In addition, repeatedly throughout the literature the presence and degree of extracranial involvement have been cited as a very important consideration in management of decision making and predictor of survival. As many of these cancers tend to be radio- and chemoresistant [1, 2], their management becomes challenging. Surgical resection of their BM can improve neurological function and is a safe, feasible option in select patients with a favorable KPS and controlled systemic disease. This intervention should be also considered for solitary brain lesions with sequelae and/or mass effect. Enhancements in surgical methods, anatomical navigation tools, brain mapping techniques, and awake neurosurgery have made it possible to excise multiple lesions in previously considered inoperable locations [1, 2].

While SR has been suggested to lead to improved outcomes [1, 2, 5, 9, 38, 144], it is not an option for many patients. When the number of metastatic foci is beyond a certain point (2-3), STRS should be considered and can provide similar or even better results [15]. This modality should be considered in those with less than 6 lesions, each < 3–3.5 cm in diameter [15]. Palliative treatment with WBRT and chemotherapy should be provided to those with many BMs and concurrent systemic disease, keeping in mind that an aggressive approach with multimodality treatment with or without SR can be judiciously given to certain patients as stated above. More recent studies have highlighted the potential of new chemotherapeutic and radiotherapeutic adjuvants [145, 146]. Overall mean survival is estimated at 7–16 months, with the majority surviving <12 months (Ewing's sarcoma, liposarcoma, fibroblastic/fibrohistiocytic tumors, malignant peripheral nerve sheath tumors, angiosarcoma, and chordomas) and a minority >12 months (osteosarcoma, chondrosarcoma, rhabdomyosarcoma, and alveolar soft part sarcoma). Bone and soft tissue BM in children is more atypical and is even more problematic to make meaningful conclusions on management and outcomes.

## 5. Conclusion

Brain metastasis in bone and soft tissue cancers usually occurs late in the malignancy. As most of the data in the literature is from case reports and case series utilizing heterogeneous treatments, it is difficult to discern the best therapeutic strategy. Prolonged survival and an adequate quality of life are achievable in a small, select population of patients. While such a survival advantage may exist for those given an aggressive treatment course involving surgical resection, it should be reserved for those with a favorable preoperative performance status and minimal systemic disease. Future large prospective studies can help give more insight on this uncommon, yet growing group of brain metastases. Overall, the occurrence of BM in patients with bone and soft tissue cancers is a poor prognostic sign that suggests late stage disease.

## Figures and Tables

**Table 1:** Comparison of published reports detailing characteristics of patients with brain metastases from primary osteosarcoma.

Case author	Age, sex	Primary site	Primary treatment	IB Mo	Metastatic site	Metastatic treatment	OS Mo	Special aspects
Rabah et al., 2013 [13]	10 F	Right humerus	(None)	0^a^	Right frontal lobe	Chemo, WBRT	14	Cerebral metastasis on presentation

Onodera et al., 2012 [14]	14 F	Left femur	Surgery, chemo	12	Right parietal lobe	SR	NR	No active pulmonary metastasis

Chou et al., 2011 [4]	*N* = 5 15–58, M (4)	NR	NR	NR	Single cerebrum (3); multiple (2)	SR + RT	D	

Flannery et al., 2010 [15]	*N* = 3 17–37, M (3)	Pelvis, femur, humerus	Chemo	NR	1 single, 2 multiple	GKSRS	36–84	Gamma knife radiosurgery as therapy

Salvati et al., 2010, 1998 [2, 9]	*N* = 10 11–60, M (5)	Femur, tibia	NR	0–110	Frontal (5), multiple (3), parietal, temporal	SR + WBRT	NR	

Niazi et al., 2009 [20]	16 M	Right femur	(None)	0^a^	Left cerebellum	SR	6 days	Posterior fossa hemorrhage on presentation

Kebudi et al., 2005 [3]	*N* = 5 12–16, M (3)	NR	NR	3–27	Single (3), multiple (2)	None (2); RT + chemo (3)	1–6	Literature review included in original study

Yonemoto et al., 2003 [18]	14 F	Right femur	SR + chemo	26	Tumor infiltration outside the bone cortex	SR + WBRT	Alive at 72	N/a

Paulino et al., 2003 [17]	*N* = 5 11–19, M (4)	Femur (2), humerus, tibia, scapula	NR	10–48	Frontal (3), occipital (1), multiple (1)	SR, chemo, WBRT, None (2)	1–10	

Weil et al., 2005 [21]	26 M	Right tibia	Chemo + SR	36	Skull and brain metastasis	SR	4	Patient refused chemo or RT for brain met treatment

Bouffet et al., 1997 [16]	*N* = 3 9-17, M	NR	NR	12–15	Supratentorial (2)	None RT (2)	2–4	

Wroński et al., 1995 [5]	*N* = 5 7–26, M (2)	Femur (3), maxilla humerus	NR	18–63	Temporal, parietal/occipital (3), posterior fossa	SR	2.4–13	WBRT was given to select patients (unspecified)

Chang et al., 1994 [22]	20 M	Bilateral femur	(None)	0^a^	Right frontal lobe	SR + RT	5	Early metastasis, multifocal

Marina et al., 1993 [19]	3 M	Left humerus	SR, chemo	4	Temporal and left parietal	Chemo	Alive at 108	Additional review of 13 patients

Wexler et al., 1993 [23]	10 F	Right femur	SR, chemo	51	Left parietal and occipital	SR, WBRT	Alive at 120	N/a

Niedeggen et al., 1990 [24]	7 M	Left femur	SR, chemo	76	Right parietal lobe	SR, RT	Alive at 13	Histological findings included

Baram et al., 1988 [25]	*N* = 5 8–15, M (2)	NR	Chemo ± SR	2–46	Frontal (2), multiple (1), NR (2)	Chemo ± SR	D	Incidence, clinical, and radiological findings and management

Lewis, 1988 [26]	15 F	Right humerus	Local radiotherapy	0^c^	Left frontal lobe, left corpus callosum	Chemo	0	Brain lesions discovered upon autopsy

Ozarda et al., 1983 [27]	23 M	Right femur	NR	8	Right occipital lobe	Chemo + RT	NR	Bone scintigraphy usage

Danziger et al., 1979 [28]	*N* = 3 18–20, F (3)	Right femur (3)	SR, chemo, RT	6–24	Temporal/occipital, frontoparietal, parietal	Chemo + RT SR, none	D (2), NR	

IB: interval to brain metastasis (mo); OS: overall survival in months; M: male; F: female; chemo: chemotherapy; SR: surgical resection; GKSRS: Gamma knife stereotactic radiosurgery. WBRT: whole brain radiotherapy; RT: radiotherapy; D: death prior to treatment; NR: not reported; ^a^brain metastasis at initial presentation; ^b^alive at last follow-up without recurrence; ^c^brain metastasis at autopsy.

**Table 2:** Comparison of published reports detailing characteristics of patients with brain metastases from Ewing's sarcoma.

Case author	Age, sex	Primary site	Primary treatment	IB Mo	Metastatic site	Metastatic treatment	OS Mo	Special aspects
Baid et al., 1992 [31]	8 M	Left thigh	RT chemo	1	Right sellar and parasellar region	WBRT	NR	No response of local disease to chemotherapy
Mineura et al., 1989 [147]	5 F	Axilla	SR chemo	5	Right occipital region	SR, WBRT, C, I	0	Suppression of blood flow, metabolism of grey matter adjacent to BM
Capitini et al., 2009 [34]	26 M	Left lateral femur	RT chemo	3	Left parietal and right occipital	SR, WBRT, GKSRS	10	Developed GVHD after allogeneic hematopoietic stem cell transplant
Simpson et al., 1989 [32]	21 M	Right 6th and 7th rib	SR chemo	24	Left parietal cortex	SR, WBRT	2^b^	Initial sarcoma misdiagnosed as costochondritis,
Parasuraman et al., 1999 [35]	*N* = 11 5–15, M (5)	Pelvis (4), humerus, scapula, tibia, vertebrae (2)	RT chemo	12–54	Parietal (4), frontal (5), temporal	RT, S, chemotherapy	3	Pediatric case series
Turgut et al., 1994 [36]	22 M	Sacroiliac joint	SR chemotherapy	24	Temporal lobe, parietal	RT	8	No active lesion within primary, BM occurred 24 months s/p treatment
Kies and Kennedy, 1978 [37]	*N* = 3 17.4	NR	RT, chemo	NR	Multiple	WBRT	3	Retrospective analysis of 134 patients with Ewing sarcoma. Found 3 BM
Olivi et al., 1991 [30]	30 F	Rib	SR. RT, chemo	48	Right posterior frontal	WBRT, S, E, Df C, D	19^b^	Pancytopenias, and *Pneumocystis carinii* pneumonia.
Chou et al., 2011 [4]	*N* = 2 17–33; M (1)	NR	RT, chemo	NR	Multiple	Palliative	NR	Respiratory failure, neuropenian, pneumonia
Salvati et al., 2010, 1998 [2, 9]	*N* =5 19–55, M (4)	Femur (2), sacrum, tibia, ulna	RT, chemo	1–13	Multiple (2), occipital, frontal, parietal	WBRT/unspecified	NR	Retrospective analysis of 35 cases of supratentorial brain metastases
Paulino et al., 2003 [17]	*N* = 6 18–20, M	Scapula (2), rib, femur, humerus, pelvis	RT, chemo	0–8	Multiple (2), frontal, parietal, temporal (2)	WBRT + chemo (5), palliative	1.5–9	1 death due to BM
Ogose et al., 1999 [33]	*N* = 2 17–44, M (1)	Thigh, buttock	RT, chemo	3-4	Right temporal, occipital	Conservative	1–6	
Bindal et al., 1994 [38]	*N* = 4 7–58, M (3)	Tibia, femur, thigh, chest wall	NR	29–115	Multiple (3), frontal	S (3),WBRT	NR	
Flannery et al., 2010 [15]	33 F	Pelvis	RT, chemo	96	Multiple	S,WBRT,GKSRS	24	
Bouffet et al., 1997 [16]	17 M	NR	RT, chemo	11	Multiple	RT	2	Patient expired secondary to BM

IB: interval to brain metastasis (mo); OS: overall survival in months; M: male; F: female; chemo: chemotherapy; SR: surgical resection; GKSRS: Gamma knife stereotactic radiosurgery. GVHD, graft versus host disease; ICH: intracerebral hemorrhage on presentation; WBRT: whole brain radiotherapy; RT: radiotherapy; D: death; NED: no evidence of disease; NR: not reported; NF: associated with neurofibromatosis type-1; ^a^brain metastasis at initial presentation; ^b^alive at last follow-up without recurrence.

**Table 3:** Comparison of published reports on patients with brain metastases from chondrosarcoma and chordoma.

Case author	Age, sex	IB yr.	BM treatment	OS Mo
Chondrosarcoma				
Flannery et al., 2010 [15]	14 M, 56 M	0.5–1.3	GKSRS	18–21
Francés-Muñoz et al., 2012 [39]	53 F	10	Chemo for lung	NR
Kawaguchi et al., 2012 [43]	54 M	0.7	Chemo	52
Jallu et al., 1992 [41]	54 F	0.7	SR	NR
Konishi et al., 1994 [44]	72 F	2	SR	2
Reyaz and Ashraf, 2006 [45]	34 F	7	SR	NR
Talerman, 1967 [46]	26 F	—	—	—
Templeton et al., 1985 [47]	15 M	34	D	NR
Waga et al., 1972 [48]	32 M	12	SR	NR
Tsutsumi et al., 2010 [40]	60 M	4.4	Chemo, GKSRS	10+
Fox et al., 1968 [49]	51 F	—	D	11

Chordoma				
Kamel et al., 2005 [53]	12 M	32	SR	32 (alive)
Anderson and Meyers, 1968 [54]	69 M	24	SR (gross total)	36 (alive)
Al-Adra et al., 2011 [52]	29 M	16	SR, WBRT	?
Morris and Rabinovitch, 1947 [56]	41 M	2.5	WBRT	2.5
Fichardt and De Villiers, 1974 [55]	59 M	36	SR	42
Higinbotham et al. 1967 [50]	58 F	?	None (autopsy finding)	72
Mesgarzadeh et al., 2008 [57]	27 F	18	None	24

Chemo: chemotherapy; D: death prior to treatment; F: female; GKSRS: Gamma knife stereotactic radiosurgery; IB: interval between diagnosis of brain metastasis from chondrosarcoma in years (yr); M: male; NR: not reported; OS: overall survival in months; Radio: radiotherapy; SR: surgical resection; WBRT: whole brain radiotherapy; ^a^diagnosed postmortem; ^b^brain metastasis diagnosed first; +: survival reported for at least the stated amount.

**Table 4:** Comparison of published reports detailing characteristics of patients with brain metastases from fibroblastic and fibrohistiocytic tumors.

Case author	Age, sex	Tumor type; primary site	Primary treatment	IB Mo	Metastatic site	Metastatic treatment	OS Mo	Special aspects/ Bone versus soft tissue origin
Wu et al., 2012 [59]	32 F	MFH; maxillary sinus	SR (radical); RT	24	Right parietal	SR; WBRT	25	*Origin: softtissue *

Graber et al., 2011 [60]	56 M	MFH; aorta	RT; chemo	0^a^	Multiple	Biopsy	?	Cystic brain lesion; mimics neurocysticercosis *Origin: softtissue (aorta) *

Louis et al., 2007 [58]	38 M	MFH; mandible	SR; RT	12	Extensive, unspecified^a^	None	12	MFH associated w/a Marjolin's ulcer *Origin: bone *

Kousar et al., 2009 [70]	20 F	AFS; mandible	SR; RT	6	Multiple	None	15	*Origin: odontic = bone *

Hoshi et al., 2008 [63]	45 M	MFH; forearm	SR; RT; chemo	48	Multiple, parietal, and occipital	Chemo, WBRT	60	Previous lung/bone metastasis at 3 yrs; SC metastasis at 5 yrs *Origin: softtissue *

Erguvan-Önal et al., 2004 [71]	45 F	FS; thigh	SR; RT	2	Right frontal	SR	7	Epitheloid transformation; *Origin: softtissue *

Rogers and Whelan, 2000 [64]	*N* = 4 40–54, M (2)	MFH; humerus, femur, knee, pelvis	Chemo; RT	4–148	Multiple (2), single (2)	RT (2), none (2)	7–153	Previous systemic metastasis in all *Origin: bone *

Ogose et al., 1999 [33]	*N* = 5 40–66, M (2)	Thigh (3), back	NR	0–10	Frontal (3), parietal, temporal	None (2), SR (2)	0.5–16	*Origin: unknown *

Salvati et al., 2010 [2]	*N* = 2 21–30, M	MFH; heart, heart	RT, chemo	19–21	Frontal	SR	9–11	*Origin: soft tissuee *

I. A. Auer and R. N. Auer, 1998 [72]	43 M	DFS; abdomen	NR	72	Tempro-parietal	SR	144	Brain recurrence at 144; prior skin, lung metastasis

Kim et al., 1997 [69]	18 F	MFS; heart	SR, RT, chemo	24	Occipital, cerebellum	SR, WBRT	50	*Origin: softtissue *

Jeffery and Ford, 1995 [67]	67 F	FS; abdominal wall	SR	60	Right parietal lobe	None	61	ICH; previous bone metastasis; *origin: sof-tissue *

Onoda et al., 1990 [73]	45 M	DFS; upper arm	SR	76	Multiple	none	84	Lungs, skin

Wroński et al., 1995 [5]	*N* = 3 28–67, M (2)	MFH; scalp, thigh, pul.a.	NR	3–27	Occipital, parietal, posterior fossa	SR	10.2^b^–65^b^	Lungs

Bindal et al., 1994 [38]	*N* = 3 25–40, M (2)	MFH; trunk, heart, shoulder		12–2218	Right parietal, multiple (2)	SR, WBRT	2.3^b^–11.8^b^	Prev lungs

Lewis, 1988 [66]	36–53, M	DFS; clavicular; MFH, scapular	SR; RT, chemo	36–312	Multiple	SR; none^c^	313, LTF	*Origin: softtissue *

Takamiya et al., 1986 [65]	43 M	FS; pectoralis major	SR	1	Right frontoparietal	SR, RT, WBRT	Alive at 13	*Origin: sof-tissue *

Zucker et al., 1978 [61]	42 F	FS; liver?	None	24	Pons	none	24	*Origin: softtissue *

Dal Canto and Valsamis, 1973 [62]	60 F	FS; renal capsule	None	0	Pons	None	0	*Origin: softtissue *

Gercovich et al., 1975 [42]	*N* = 2 31–52 M	FS; lip, retroperitoneal	Chemo	3–55	Frontal, parietal	NR	NR	*Origin: softtissue *

Ho, 1979 [68]	70 F	FS; thigh	SR (partial)	12	Leptomeninges, cortical	None^c^	NR	*Origin: softtissue *

AFS: ameloblastic fibrosarcoma; FS: fibrosarcoma; DFS: dermatofibrosarcoma; IB: interval to brain metastasis (mo); OS: overall survival in months; M: male; F: female; chemo: chemotherapy; GKSRS: Gamma knife stereotactic radiosurgery; MFS: myxofibrosarcoma; ICH: intracerebral hemorrhage on presentation; SR: surgical resection; WBRT: whole brain radiotherapy; RT: radiotherapy; D: death prior to treatment; LTF: lost to follow-up; MFH: malignant fibrous histiocytoma (undifferentiated pleomorphic sarcoma); NR: not reported.

^a^Brain metastasis diagnosed at presentation; ^b^survival after craniotomy; ^c^brain metastasis reported at autopsy.

**Table 5:** Comparison of published reports detailing characteristics of patients with brain metastases from liposarcoma and rhabdomyosarcoma.

Case author	Age, sex	IB yr.	BM treatment	OS Mo
Rhabdomyosarcoma				
Ahola et al., 1998 [84]	13 M	0.7	SR	14
Andersen-Ranberg and Helmer-Hansen, 1987 [83]	19 M	0.5	NR	6+
Flannery et al., 2010 [15]	18 F	5.0	GKSRS, WBRT	60
Ho, 1979 [68]	79 M	—	D	—
Kebudi et al., 2005 [3]	11 M	0	RT, Chemo	3
Kleinert et al., 1985 [90]	14 F		D	48
Noda et al., 1995 [91]	2 M	0.5	Chemo, RT	32+
Ogose et al., 1999 [33]	7 F, 16 M	NR, 0.2	Chemo, RT	4, 4
Osawa et al., 2011 [88]	*N* = 3 6–18, M (3)	1.1–12.2	SR, RT, Chemo	21–207
Paulino et al., 2003 [17]	*N* = 8 1.7–18, M (6)	NR	WBRT, Chemo	2–63
Rodriguez-Galindo et al., 2001 [85]	*N* = 5 Newborn, F	0.1–1.1	NR	1.5–24
Salvati et al., 2010 [2]	*N* = 2 20–22, M (1)	0.2–0.3	SR	12.8

Liposarcoma				
Arepally et al., 1996 [76]	56 M	312	SR, WBRT	324
Bailey et al., 2001 [74]	54 F	108	SR, WBRT	Alive at 114
Can et al., 1993 [82]	22 M	0	none	
Ferguson et al., 2006 [79]	48 F	276	RT	278
Fitzpatrick et al., 1999 [77]	74 F	24	SR	Alive at 30
Haft et al., 1988 [80]	52 F	216	SR (total), chemo	228
Kumar and Teasdale, 2000 [81]	73 F	12	SR	Alive at 12
Salvati et al., 2010 [2]	48 M	NR	SR	NR
Utsunomiya et al., 1999 [75]	44 M	72	SR (subtotal)	77

IB: Interval to brain metastasis (mo); OS: overall survival in months; M: male; F: female; chemo: chemotherapy; SR: surgical resection; GKSRS: Gamma knife stereotactic radiosurgery; ICH: intracerebral hemorrhage on presentation; WBRT: whole brain radiotherapy; RT: radiotherapy; D: death prior to treatment; NR: not reported; UK: unknown; ^a^survival after craniotomy.

**Table 6:** Comparison of published reports detailing characteristics of patients with brain metastases from malignant peripheral nerve sheath tumor (MPNST) and angiosarcoma.

Case author	Age, sex	IB yr.	BM treatment	OS Mo
Malignant peripheral nerve sheath tumors				
Xu et al., 2012 [93]	8 M	14	(None)	16
Flannery et al., 2010 [15]	34 F	36	SR, WBRT, GKSRS	48
Tilgner et al., 2007 [92]	60 M	0^a^	SR, WBRT	Alive at 14
Park et al., 2007 [101]	21 M	0^a^	SR, WBRT, Chemo	16
van Eck and Horstmann, 2006 [100]	83 M	108	SR, GKSRS	122
Matyja et al., 2004 [94]	33 M	~56	SR	~60
Yone et al., 2004 [104]	4 M	7	WBRT	21
Oishi et al., 2000 [102]	48 M	61	SR (en bloc)	NR
Maschke et al., 1999 [105]	17 F	0^a^	SR	NR
Probst-Cousin et al., 1997 [106]	19 F	156	None	157
Haisa et al., 1996 [103]	58 F	180	SR (en bloc)	181
Fenzi et al., 1995 [99]	45 F	5	None	18
Seppälä and Haltia, 1993 [107]	13 M	2	None	2
D'Angelo et al., 1991 [115]	68 F	24	SR (en bloc)	36
Valdueza et al., 1991 [108]	47 M	~13	None	18
Cras et al., 1990 [109]	46 F	0	None	0
Hirose et al., 1989 [110]	53 M	15	SR (en bloc), chemo	Alive at 20^b^
Hasegawa et al., 1984 [111]	51 M	2	Biopsies	3
Macaulay, 1978 [112]	18 M	2	None	2
White Jr., 1971 [113]	20 M	NR	SR, radiation	7
D'Agostino et al., 1963 [114]	22 F	NR	NR	68

Angiosarcoma				
Liassides et al., 2004 [119]	24 F	3	Palliative	6
Eguchi et al., 2002 [123]	64 M	19	Palliative	0
Chami et al., 1994 [118]	59 M	NR	Palliative	NR
Vaquero et al., 1990 [124]	30 M	4	SR	6
Kuratsu et al., 1991 [117]	17 M	—	SR	12
Haft et al., 1988 [80]	31 F	—	WBRT	NR
Akutsu et al., 2004 [125]	53 M	2	SR	6
Ellegala et al., 2002 [121]	76 M	24	SR	2
Søndenaa et al., 1993 [126]	73 F	3	Palliative	3
Gallo et al., 2001 [127]	33 M	12	SR	0
Hassane et al., 2010 [128]	48 M	3	Palliative	0
Simpson et al., 1989 [32]	21 M	24	SR, WBRT	26^b^
Angrish et al., 1979 [120]	38 M	3	Palliative	0
Plotnik et al., 2008 [122]	61 F	60	SR	NR
Chaudhuri et al., 1980 [129]	31 F	72	SR, WBRT	2
Macaulay, 1978 [112]	18 M	2	Palliative	0

IB: interval to brain metastasis (mo); OS: overall survival in months; M: male; F: female; chemo: chemotherapy; SR: surgical resection; GKSRS: Gamma knife stereotactic radiosurgery; ICH: intracerebral hemorrhage on presentation; WBRT: whole brain radiotherapy; RT: radiotherapy; D: death prior to treatment; NR: not reported; NF: associated with neurofibromatosis type-1; ^a^brain metastasis at initial presentation.; ^b^alive at last follow-up without recurrence.

**Table 7:** Summary of cases of alveolar soft part sarcoma with brain metastases as reported in previous published series.

Case Author	*N*	Age, sex	Primary site	Primary treatment	IB Mo	*N* with BM	Metastatic treatment*	OS* (Mo)	Special aspects
Daigeler et al., 2008 [130]	11	19–24, M (7)	Thigh (2), lower leg (2), thoracic wall (2), upper arm (2), forearm (1), Foot (1)	SR; chemo with RT	78 months (5–156)	3/11	SR, RT	73% of patients are still alive at follow-up with no evidence of disease	Localized disease, complete resection. And adjuvant radiation within 2 years had favorable outcome

Kayton et al., 2006 [138]	20	16.5; 6–24, M (10)	Thigh (8), trunk (6), Retroperito. (2), scalp, neck, forearm, calf	SR, chemo, RT	36 months	2/20	SR	Follow-up: patients were alive after mean 22 years (4–32 years)	Tumors, 5 cm were associated with longer progression-free survival

Park et al., 1999 [139]	6	24.5 (17–35) (2) M	Femur (3), fibula (2), ilium	SR, chemo, RT	10–12 months	2/6	N/A	Follow-up range of patients with no evidence of disease (7 mo.–8 yrs.)	ASPS arising in bone

Portera et al., 2001 [134]	74	26 (3–68) 49% male	Extremities (60%), trunk (20%), head and neck (12%), Retroper. (8%)	SR; RT; chemo	5–24 months	14/74	Chemo	Median was 41 months for patients w/o metastasis and 40 months with metastasis	

Bindal et al., 1994 [38]	2	40–48 1 male	Thigh, rectum	SR	1–15 months	2/2	SR	Still alive after 24.7 and 16.4 months	

Flannery et al., 2010 [15]	2	42.4 (14–74)	Leg, gluteal	Chemo and SR	30–48 months	2/2	SR	Survived from 5 to 7 years after surgery with no evidence of disease; 31–36 months after metastasis	

Salvati et al., 2010 [2]	3	35 (19–61); males	Thigh (100%)	SR	24–58 months	3/3		Alive from 15 to 24 months after SR with no evidence of disease	

Wroński et al., 1995 [5]	2	7–14 1 male	Thigh, tongue	SR	23 months	2/2	SR	Alive 2.7–23 months SR with no evidence of disease	

Chou et al., 2011 [4]	4	25 (17–33); M	NR	SR and RT	14.53 months	4/4	SR and RT	3 dead; 1 still alive	

*Some results are reported for an entire series of ASPS patients, and not necessarily only those with BM; ASPS: alveolar soft part sarcoma; BM: brain metastasis; IB: interval to brain metastasis (mo); OS: overall survival in months; M: male; F: female; chemo: chemotherapy; SR: surgical resection; GKSRS: Gamma knife stereotactic radiosurgery; ICH: intracerebral hemorrhage on presentation; WBRT: whole brain radiotherapy; RT: radiotherapy; D: death prior to treatment; NR: not reported; UK: unknown; ^a^survival after craniotomy.
